# Analysis of Building Envelope Insulation Performance Utilizing Integrated Temperature and Humidity Sensors

**DOI:** 10.3390/s120708987

**Published:** 2012-06-29

**Authors:** San-Shan Hung, Chih-Yuan Chang, Cheng-Jui Hsu, Shih-Wei Chen

**Affiliations:** 1 Department of Automatic Control Engineering, Feng Chia University, No. 100, Wenhwa Road, Seatwen, Taichung 40724, Taiwan; E-Mail: k784101@hotmail.com; 2 Department of Civil Engineering, Feng Chia University, No. 100, Wenhwa Road, Seatwen, Taichung 40724, Taiwan; E-Mails: rchang@fcu.edu.tw (C.-Y.C.); show.1717@hotmail.com (C.-J.H.)

**Keywords:** integrated sensor, temperature, humidity, reinforced concrete (RC), energy management, building envelope, wireless communication

## Abstract

A major cause of high energy consumption for air conditioning in indoor spaces is the thermal storage characteristics of a building's envelope concrete material; therefore, the physiological signals (temperature and humidity) within concrete structures are an important reference for building energy management. The current approach to measuring temperature and humidity within concrete structures (*i.e.*, thermocouples and fiber optics) is limited by problems of wiring requirements, discontinuous monitoring, and high costs. This study uses radio frequency integrated circuits (RFIC) combined with temperature and humidity sensors (T/H sensors) for the design of a smart temperature and humidity information material (STHIM) that automatically, regularly, and continuously converts temperature and humidity signals within concrete and transmits them by radio frequency (RF) to the Building Physiology Information System (BPIS). This provides a new approach to measurement that incorporates direct measurement, wireless communication, and real-time continuous monitoring to assist building designers and users in making energy management decisions and judgments.

## 1. Introduction

Recently, energy consumption and management issues have received significant attention from governments, industry, and academia. Previous research [1] showed that commercial and residential buildings account for approximately one-third of global energy consumption. The lifespan of a building is greater than that of other industrial products; therefore, the accumulated effect of energy conservation in buildings is also greater than that of other industries [2]. Among newly constructed buildings in Taiwan, 77.6% are constructed with reinforced concrete (RC) [3]. In tropical regions, RC buildings store an extremely high amount of thermal energy during the day; however, there is insufficient time for the mitigation of thermal energy during the evening. Therefore, a large amount of air conditioning systems are used to maintain comfortable living conditions, resulting in greater energy consumption [4]. Using the calculations of the air conditioning cooling load, more than 30% of building cooling load is caused by the thermal energy of solar radiation that enters through envelope walls and windows [5]. Therefore, using insulation in the building envelope is one of the most effective methods for building energy conservation, thereby reducing the adverse effect of building envelope thermal storage. Furthermore, if the internal temperature of concrete could be monitored instantly when it absorbs significant amounts of radiated heat (*i.e.*, roof tops and outer walls with southern and western exposures) and combined with the building's own temperature self-regulating actions, then air conditioning load can be reduced efficiently and comfortable indoor conditions can be maintained.

Currently, several technologies and studies use sensors to measure temperature and humidity in the atmosphere. Previous research [6] employed a wireless weather monitoring system using micro-electro-mechanical systems (MEMS) and wireless sensor network (WSN) technologies to monitor temperature, humidity, air pressure, flow rate, and direction. However, for temperature and humidity monitoring inside buildings, there is an ongoing demand for small and reliable electronic sensors capable of monitoring water content in civil engineering materials and structures because excess water may accelerate degradation [7]. To protect critical building assets effectively previous research [8] assessed the performance of a microclimate monitoring system implemented for the preventive conservation of Renaissance frescoes in the apse vault of the Cathedral of Valencia (Spain) that were restored in 2006. This type of long-term temperature and humidity measurement facilitates effective building maintenance and management.

Furthermore, previous research [9] employed a K-type thermocouple to measure the temperature at a central point on a concrete structure and the surface temperature to understand the insulation performance of insulation material for reducing building energy usage. Other studies used formulae to infer the thermal transmittance U-value, the thermal resistance R-value, and the temperature gradient to assess its insulation performance [10]. A numerical model was also used to assess the thermal performance of building envelopes to adjust their design [11]. These methods can measure or calculate the internal temperature of concrete and the insulation performance; however, when using a thermocouple to measure the internal temperature of concrete, complicated wiring is an issue that must be addressed. For numerical modeling, actual construction work must be considered because this approach is not a homogeneous layer concept. (*i.e.*, the outer wall includes the outer wall's ornamental material, the plaster layer, the concrete layer, the steel frame layer, and the filler.) Therefore, if the temperature and humidity variation within the concrete can be directly and continuously observed at numerous locations, it can provide an enhanced reference value for designers and managers.

Therefore, using radio frequency integrated circuits (RFIC) combined with temperature and humidity sensing chips as part of an electronic circuit design, this study produces a smart temperature and humidity information material (STHIM). This is a complete package composed of RFIC and temperature and humidity (T/H) sensors that can automatically, instantly, and continuously emit a signal for a set time. The STHIM can be embedded into the building structure when pouring concrete to overcome the material characteristics of concrete's alkalinity, water permeability, and pulp fluidity. Currently, this study has successfully measured the dynamic temperature changes inside a simulated concrete structural model and can compare the performances of various insulation materials. This study also observed various degrees of influence by weather factors and endothermic and exothermic rates of various insulation materials [12]. The radio frequency (RF) communication technology addresses the issue of transmission blockage in concrete structures, and the information can be accurately recorded in detail to the Building Physiology Information System (BPIS) for long-term observation of temperature changes within concrete structures.

## 2. Building Sensors

### 2.1. Building Temperature and Humidity Integrated Transducers

Detection with a humidity sensor uses sensing material to absorb water vapor or to detect changes in electrical impedance or capacitance caused by evaporation. Humidity sensor components are divided according to their materials of construction into ceramic humidity sensors, polymer thin film humidity sensors, and electrolyte sensors. They can also be divided into two primary types according to their output signals: capacitance and impedance types. For example, previous research [13] used a resistance temperature detector (RTD) and a capacitance humidity detector combined with a high-power LED to measure the temperature and humidity of high-power LED chips. Other research [14] employed a resistance T/H sensor to measure the air temperature and humidity and a Fe_2_O_3_ gas sensor to measure and calculate the H_2_ and CH_4_ concentration in the air. These studies used common and stable humidity sensor components and achieved good results according to expectations. Therefore, this study also used the capacitance-type humidity sensor components similar to those used in [13].

Temperature sensing components use changes in air pressure, size, kinetic energy, and resistance after the temperature of an object changes to infer the temperature of the object by the relationships of these changes. However, in actual application, factors such as measurement range, accuracy, the object being measured, the environment, and cost also require consideration. Currently, the components used commonly in temperature measurement studies are thermocouples, IC temperature sensors, resistance temperature detectors (RTD), and thermistors [15–18]. These components are primarily used to measure atmospheric temperature changes or in micro-electro-mechanical systems (MEMS). These temperature sensing components are widely used in all types of applied research, although the adverse environment inside concrete with strong alkalinity and water permeability require consideration because this study must embed the components inside concrete for temperature measurement. Therefore, this study does not use common temperature sensing components. The limitations of using these components are shown in Table 1.

### 2.2. Component Selection

The sensor module used in this study can monitor the temperature and humidity within concrete for a long, continuous period (*i.e.*, it can continuously monitor these parameters for at least one year or more) [19]. The components used for this module include a T/H sensor chip, wireless communication components, and a microcontroller. The reasons for this selection are detailed as follows.

There are various types of temperature and humidity integrated sensors, and some of the sensors introduced in Section 2.1 require the design of a precision amplifier circuit, whereas others that have non-linear output characteristics require the design of a signal processing circuit to linearize the detected signal. If these T/H sensor components were used, the size of the sensor module would increase considering the complicated electronic circuit design and RFIC integration, thereby affecting the safety of concrete structures. Therefore, this study uses the SHT series of digital T/H sensors (Sensirion Company, Staefa, Switzerland). The SHT 1x–7x series is a high-precision temperature and humidity sensing chip that provides digital output. Its sensing components include an aggregate capacitance temperature and humidity sensing component and a chip that combines a bondgap 14-bit A/D converter and a serial communications interface circuit. Its calibration coefficients are stored in the one-time programmable (OTP) memory. When these coefficients are used for measurement, they are used to correct the sensor component's signal. This product has the advantages of extremely fast reaction time, excellent anti-jamming capability, small size, and low power consumption Because of the discussed advantages, several studies have adopted SHT 1x–7x series temperature and humidity sensors [20,21]. The XBee Series 2 (MaxStream under Digi International) was used for the wireless communication components. This module is an IEEE 802.15.4 stack (based on ZigBee) and uses a simple serial command to execute the ZigBee communication protocol on 802.15.4. It also supports the three modes of coordinator, router, and end device as well as the three network topologies of peer-to-peer, point-to-point, and mesh, and operates on an ISM 2.4 GHz band. Its maximum transmission range is 100 m, and its transmission speed can reach a maximum of 240 kbps. It is commonly applied in low-cost, low-power wireless sensor networks. The information transmission flow of XBee commences from the D_IN_ pin's reception of the microcontroller signal, and the data is stored in a T_X_ register until all information is received. After the microcontroller's information is received, the RF switch is activated to switch to transmission mode. Using the transmitter, the information in the T_X_ register is communicated wirelessly to the XBee receiver. The receiver converts the received information and stores it in the R_X_ registry until all information is communicated. The information in the registry is transmitted to the microcontroller or computer through the D_OUT_ pin for processing.

This study uses a C8051F410 microcontroller (Silicon Lab, Austin, USA), that has a built-in A/D converter (ADC), UART interface, SIP interface, and comparator functions. The operating voltage is 2.0 to 5.25 V, and the built-in 24.5 MHz tunable oscillator can also be connected externally. The unit also has a suspend mode, RTC interrupt and wakeup settings. The system primarily uses RTC interruption to wake up the transmitter system's suspension, a UART interface for communications between the controller and the RF module, a timer, and pin control sensors. It uses C programming language.

**Figure 1.**
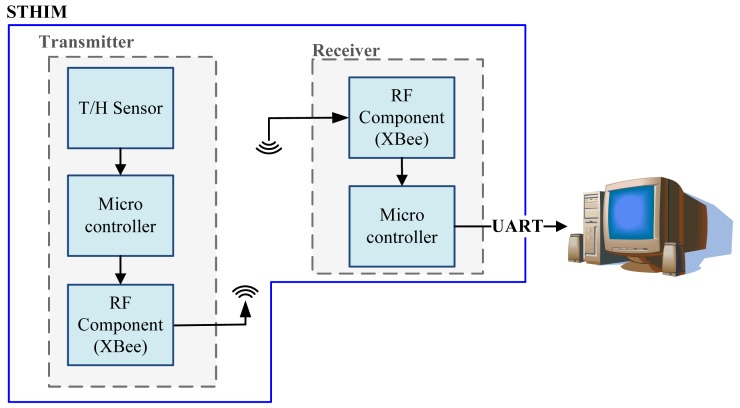
STHIM system block diagram.

**Figure 2.**
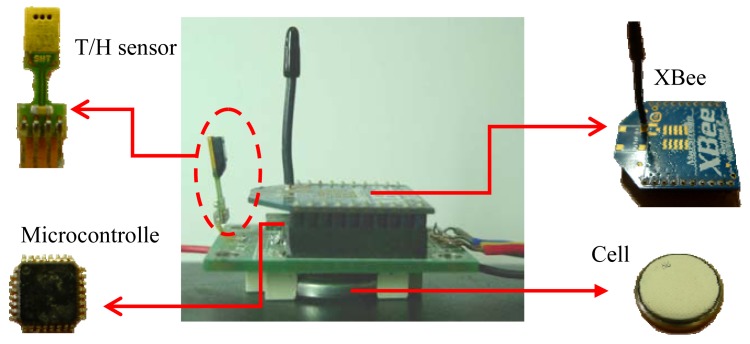
STHIM transmitter actual configuration.

This system architecture can be divided into two main sections: the transmitter and the receiver, (the same system architecture was adopted in previous research in [21]). The transmitter uses the internal ADC to convert the temperature and humidity signal detected by the T/H sensor into digital signals, and use microcontroller to coding these signal. The signal is then sent through the XBee to the receiver. The receiver microcontroller decodes the information and uses the communications protocol (UART) to transmitit to the computer, where the received data is displayed on the monitoring interface, (Figure 1). The actual configuration of the transmitter is shown in Figure 2.

The key STHIM experimental settings primarily investigate the design of the STHIM transmitter encapsulation box during the research and development process. The STHIM transmitter must be embedded in concrete to measure the internal temperature and relative humidity of reinforced concrete, and a 3D space must be constructed for the RH/T sensor to allow the measurement of temperature and relative humidity. However, freshly mixed concrete has strong alkalinity, and water in concrete and grouting impact can damage STHIM transmitters. Therefore, the design of the STHIM transmitter encapsulation box requires multiple experiments and modifications during the research and development process. Following several rounds of experiments and discussions, the research team has solved problems concerning the design issues of the encapsulation box, including the construction of a 3D measuring space inside the encapsulation box, size reduction, impact-endurance or resistance, prevention of electromagnetic interference, water-resistance, and outer covering motar (including the propagation of temperature and humidity) [19].

### 2.3. User Interface

In [22] the graphical user interface is a Java-based program operated from a host computer. Users could add a new patient to the monitoring network by keying in the patient name and the ID of the patient's assigned sensor node. Once entered into the system, all readings from the patient's sensor node are stored as object variables corresponding to that patient. All stored readings can be viewed by selecting a patient name from a list and then selecting the “view” option. In [23], the interface presents the last recorded values of the solar total, diffuse, and calculated direct radiation intensities and offer the indicated in the figure, selection criteria options, for the recorded data. The potential time step values (depending of the selected criteria) are 1, 5, 10, 15, 20, 30, 60 min. Each selection criteria is activated by pressing one of the buttons marked by “list recorded values.” The features and functions of the system user interface can be observed in the discussed studies to vary according to different measurement objects and goals.

To design a friendly and clearly user interface to users like [22,23] is necessary. The user interface in this study (Building Physiology Information System, BPIS) uses Borland C^++^ Builder (BCB) interface software for database development. The monitoring interface examined the point numbering, monitoring point temperature and humidity data, time of data reception, statistics, and database establishment.

**Figure 3.**
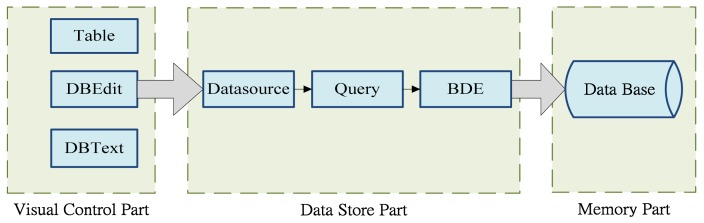
Conceptual diagram of BCB database element connections.

The functional components of the C^++^ Builder's database wizard and database desktop provided the overall database's software writing for connection settings. The connections between the database elements primarily used the display control components necessary for object control options, such as visualization components like Table, DBEdit, and DBText. This study uses the data source data storage component to transfer, access, and connect to other databases for processing. Finally, we used Borland Database Engine (BDE) to access data directly for database building. The conceptual diagram of BDE is shown in Figure 3.

**Figure 4.**
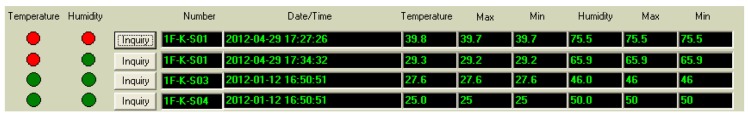
Temperature and humidity monitoring display.

**Figure 5.**
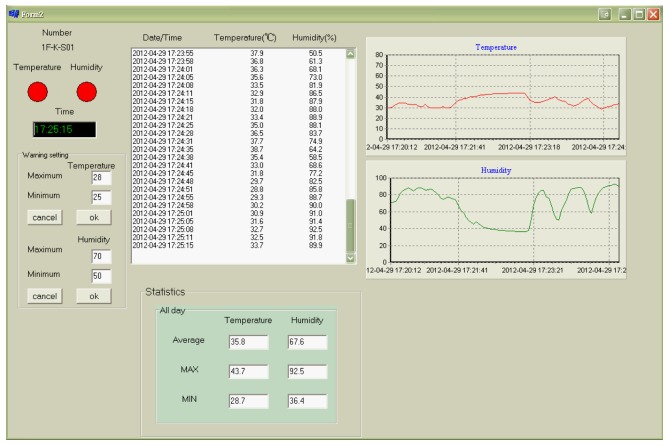
Display of point monitoring.

Figure 4 shows the instant temperature and humidity data for each monitoring point. After the signal received by the receiver's wireless communication module is processed, it immediately displays the current reception time, temperature, humidity, maximum received temperature, and minimum received humidity values. If the received temperature and humidity values exceed the pre-set upper and lower limits, the warning indicator turns red, as shown in Figure 5. The inquiry button provides detailed real-time data for specific point.

The information shown in Figure 5 includes a warning indicator, warning occurrence time, maximum/minimum temperature and humidity settings for each monitoring point, reception time, temperature value, humidity value, temperature/humidity dynamic graphs, and statistics (average, maximum, and minimum).

The BPIS can perform multiple-point monitoring, real-time and long-term monitoring, as well as provide value and graphical information, point anomaly settings, and anomaly warnings to assist in temperature and humidity analysis of a building's RC structure, thereby providing references for health diagnosis or maintenance management. For example, if the humidity in five monitored points on a roof panel was significantly high and humidity always fluctuated at one of these five points, this would indicate that the fluctuating point was the source of a leak. Therefore, the STHIM monitoring information assists building maintenance in pinpointing the leak accurately [24]. For temperature monitoring and management, local temperature reduction can be performed for a wall or panel that accumulates high temperature because of sunshine or other factors, thereby assisting in the reduction of energy consumption. Intelligent living space planning is a novel trend, and building self-health management is gradually becoming computerized and automated. Therefore, to effectively maintain the structural functions of a building and extend its lifespan, a self-monitoring system plan helps long-term accumulation and storage of monitoring information to provide reference values, and save significant amounts of monitoring manpower and time.

## 3. Experimental Section

The STHIM can simultaneously measure temperature and humidity in the atmosphere and within concrete and provides practical value for maintenance management. Core temperature and ambient humidity are critical factors when measuring humidity within concrete and their consideration is crucial for calculating and predicting the early strength development [25]. Humidity can also directly influence water that enters and exits concrete; the shrinkage amount is greater in environments with lower humidity. Most drying shrinkage prediction models include relative humidity as a major parameter [26]. Theoretically, temperature and humidity gradients can influence outer walls; however, calculations of water storage and transportation are often neglected for thermal transmission calculations for outer walls [27]. Temperature variations can cause shrinkage and creep on building structures, resulting in cracking and erosion of concrete structures, pre-stressed losses in pre-stressed structures, pre-stressed redistribution, and increased deformation [28]. Although STHIM can simultaneously detect the temperature and humidity within concrete, applications and measurements of humidity are not conducted at an advanced level because of funding restrictions. However, advanced experiments and analysis will be conducted when sufficient funding is obtained in the future. Currently, this research employs the limited funding that can be obtained at the present time to focus on energy-related temperature monitoring. Changes in temperature also facilitate the assessment of the concrete quality. However, an ultra-small sensor system also has a highly practical value for improving energy conservation and reducing emissions because the thermal storage characteristics of the concrete material of building envelopes are one of the primary causes of indoor spaces to require significant amounts of electricity for air conditioning. Therefore, this study prioritized energy management in the STHIM experiment planning stage and focused on the monitoring and assessment of building envelopes (e.g., walls and roofs) thermal storage.

### 3.1. Experiment Methodology and Locations

**Figure 6.**
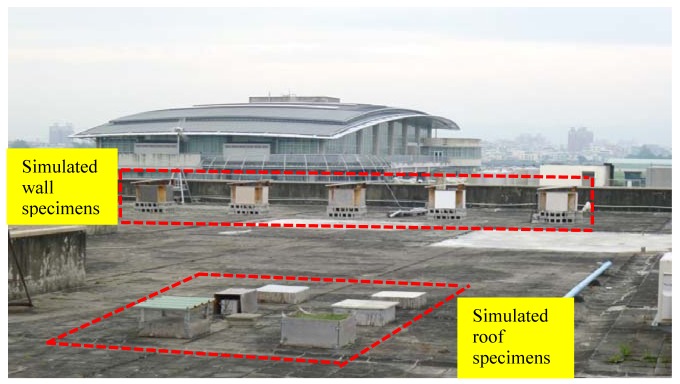
Experiment site.

This study used STHIM to measure the temperature inside concrete and compares the insulation performance of commonly used insulation materials on the roofs and walls of buildings in Taiwan. Concrete samples (50 × 50 × 15 cm) were built to simulate roof and wall structures and one STHIM was inserted into the center of each sample. To prevent the external thermal environment from interfering with the accuracy of the sample's internal temperature measurement, white Styrofoam panels (1.5 cm thick) were placed around the four sides and the bottom inside the samples to prevent external temperatures from influencing the STHIM-measured values. To ensure that the insulation material placed in the RC samples would receive solar radiation evenly and to prevent influences by accidental damage and the surrounding environment, a site situated in a wide open area is required for the experiment. The selected site was the roof of Taichung City's Feng Chia University Civil and Hydraulic Engineering Building (coordinates 120.646889E and 24.180974 N), as shown in Figure 6.

### 3.2. Simulated Roof and Wall Insulation Performance Experiment Planning

**Figure 7.**
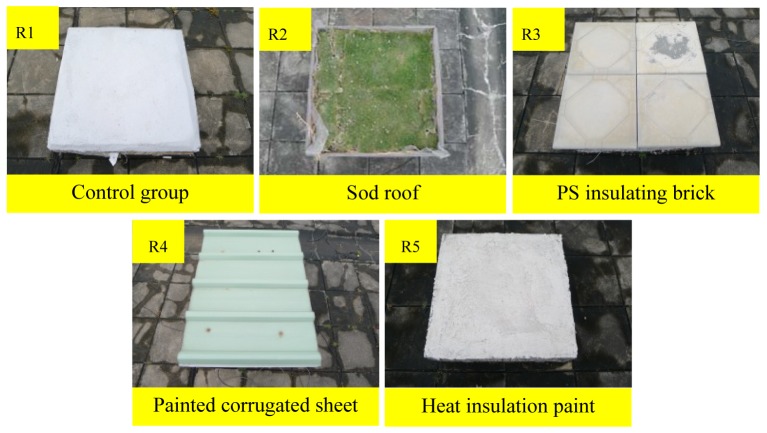
Simulated roof insulation material samples.

To compare the performances of roof insulation materials, this study designed and produced five RC samples to simulate roof structures. R1 is the control group (no insulation material) and the insulation material experimental groups are R2 (sod roof), R3 (PS insulating brick), R4 (painted corrugated sheet), and R5 (heat insulation paint), as shown in Figure 7. By continuously measuring the dynamic internal temperature variation of the samples and comparing their temperature differences with the control group, this study assesses the insulation performance of four commonly used insulation materials.

**Figure 8.**
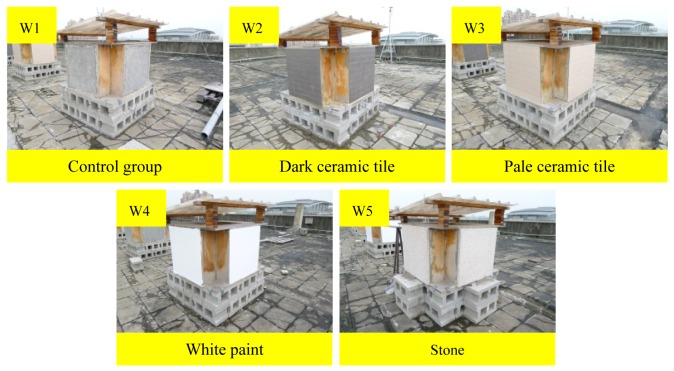
Simulated wall insulation material samples (dark and pale ceramic tile, white paint, and stone).

To compare the performances of wall insulation materials, this study designed and produced five RC samples to simulate wall structures. W1 is the control group (no insulation material), and the experimental groups are W2 (dark ceramic tile), W3 (pale ceramic tile), W4 (white paint), and W5 (stone), as shown in Figure 8. The control group and the experimental groups were composed of four simulated wall plates that faced the four cardinal directions. This study examined the temperature differences of different sunshine directions simultaneously. This study assessed the performances of the insulation materials by continuously measuring the dynamic internal temperature variation of the samples and comparing their temperature difference with the control group.

## 4. Results and Discussion

### 4.1. Roof Insulation Measurement

#### 4.1.1. Roof Insulation Performance in Different Weather Conditions

**Figure 9.**
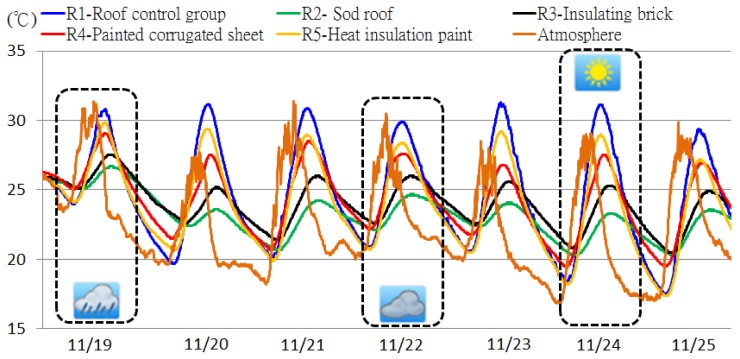
Efficacy measurements of roof thermal insulation materials.

We embedded the STHIM into the RC samples and measured their internal temperature fluctuations. The STHIM measurement frequency was set as one measurement/transmission every 5 min. The observed values from 19 to 25 November 2011 are shown in Figure 9. We also placed one STHIM layer in the roof experimental area for simultaneous measurement of outside atmospheric temperature changes during the observation period. The average outdoor atmospheric temperature during this period was 22.5 °C and there were various changes in weather patterns, including sunny, cloudy, and rainy days. For example, the sample data on 24 November was from a sunny day 22 November was a cloudy day sample, and 19 November was a rainy day. The thermal performances of all groups over the three sample days were examined. Table 2 shows the analysis of the maximum temperatures of the experiment groups R2 to R5 in comparison with the R1 control group, including the three varying weather conditions.

From these results, there was a clear insulation effect observed during the sunny day with the insulated experimental groups. The next most efficient results were observed on the cloudy day, whereas the insulation effect of the insulation material on the rainy day was not as significant. In late November (almost winter), the performance of the insulation materials was: R2 sod roof > R3 PS insulating brick > R4 painted corrugated sheet > R5 heat insulation paint > R1 no insulation material. However, these results do not indicate a year-round case. Long-term observation data is required to examine the differences in insulation material performance. However, preliminarily results confirm that STHIM can continuously and effectively measures roof sample temperature changes and the accumulated monitoring data can be a basis for future energy management strategies.

#### 4.1.2. Influence of Roof Insulation Material on Concrete Thermal Storage Characteristics

**Figure 10.**
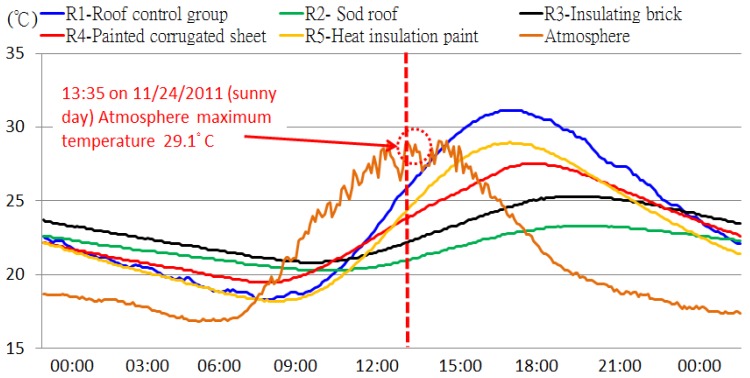
The effect of roof thermal insulation materials on heat-storing properties of concrete.

In contrast to the sensitive temperature changes measured by STHIM in the atmosphere, there was a slow rise and fall of temperature within the RC samples. This indicates the thermal storage characteristics of the RC structure and its ability to delay thermal absorption and radiation. At 13:35 on 24 November (sunny day sample), the maximum observed outdoor atmospheric temperature was 29.1 °C. The temperature for R1 (control group), R2 (sod rood), R3 (PS insulating brick), R4 (painted corrugated sheet), and R5 (heat insulation paint) was 28.3 °C, 21.5 °C, 22.9 °C, 25 °C, and 26.6 °C, respectively (Figure 10).

The observed maximum temperatures, times that maximum temperatures occurred, and the duration that the maximum temperatures were maintained were different for all RC samples. The change of temperature within the RC was influenced by the insulation material performance, the weather, and the concrete thermal absorption rate. Without being able to directly measure the dynamic changes using the STHIM, characteristics and trends are difficult to assess. For example, the maximum temperature for the R1 control group remained at 31.1 °C from 15:40 to 16:25 on 24 November (sunny day) for a continuous maximum temperature for 45 min. The R2 sod roof maintained the maximum temperature for as long as 90 min, although this temperature was only maintained for 5 min in the atmosphere. The delay time difference was as high as 18 times (Table 3). This data was measured in late autumn; therefore a greater temperature difference is anticipated for the summer.

### 4.2. Wall insulation Measurement

#### 4.2.1. Influence of Sunshine and Direction on Concrete Wall Thermal Storage

**Figure 11.**
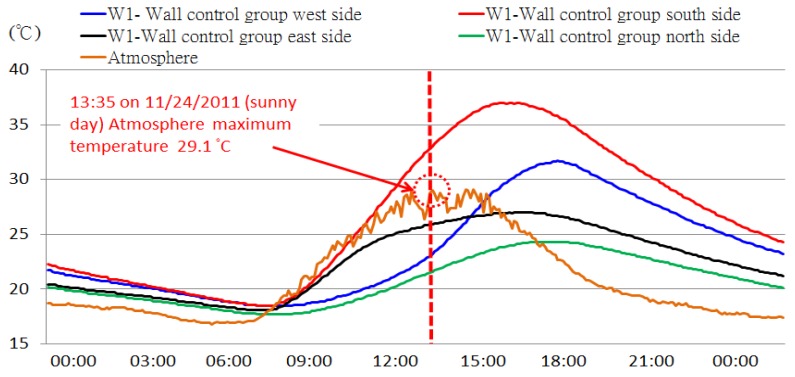
The effect of sunshine and direction on heat storage of outer walls.

The sun and the earth move in relationship to one another in an orderly pattern, so each day is divided into day and night, and each year is divided into four seasons. The angle that the sun hits a building during the day also changes constantly; therefore, degrees of thermal absorption from the sun on the walls on different sides of a building are not identical. We used the STHIM embedded in simulated wall RC samples with the W1 control group as the object of this study, and measured the dynamic changes of temperature within the wall samples facing different directions. Figure 11 shows the W1 control group on 24 November 2011 (sunny day) as an example and examines the influences of sunshine and direction on concrete wall thermal storage.

In the experimental area, the atmospheric temperature typically reaches the daily maximum temperature at approximately 13:30; however, the W1 west side sample's internal temperature did not reach the daily maximum temperature until after 16:30, with a time difference of approximately 3 h. The primary reason for this is the change in the angle of the sun. The west side takes the longest to reach the high point followed by the north, east, and south sides. The side with the highest thermal storage is the south side. The primary reason for this is that the south side has the longest duration of sunshine during the winter. The next longest are the east and west sides. The north side receives very little direct sunlight throughout the day. The observed results were consistent with astronomical principles. The details are shown in Table 4.

#### 4.2.2. Influence of Wall Insulation Material on Concrete Wall Thermal Storage Characteristics

**Figure 12.**
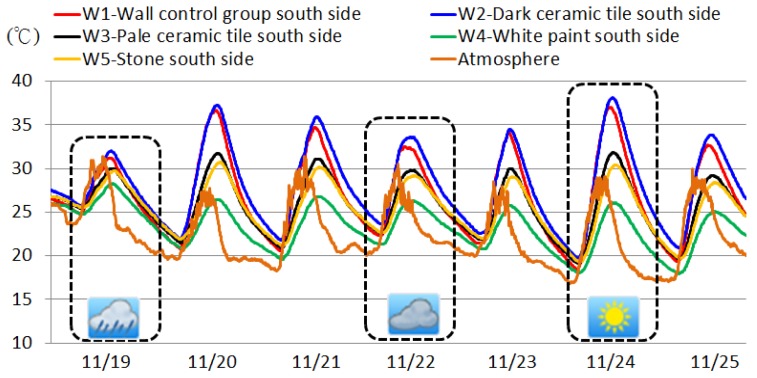
Effects of weather on thermal insulation efficacy of outer wall insulation materials.

Different insulation materials produce different effects when used to insulate roofs, and similarly, the same type of insulation effect is achieved for a building's vertical walls. Figure 12 shows the continuously observed data from 19 to 25 November, 2011. The south side receives more sunlight than the other three sides during the winter; therefore, the south side samples on a sunny day, cloudy day, and a rainy day were examined. For example, the data on 24 November was a sunny day sample, the data on 22 November was a cloudy day sample, and the data on 19 November was a rainy day sample. We used the data from the three samples to examine the insulation performance of the south sides for the W1 control group and insulated W2 to W5 experimental groups.

Table 5 shows the comparisons of measured results from the simulated outer southern wall samples under different weather conditions. Among the wall insulation materials, the paint on the walls is more effective at insulating heat, except for the white paint, which is more reflective because the current usages of building wall ornamental materials emphasizes aesthetic over insulation efficiency. However, dark ceramic tiles do not effectively insulate heat primarily because dark materials are more likely to absorb heat.

From these results, there was a clear insulation effect observed on the sunny days with the insulated experimental groups. The next most efficient was observed on the cloudy day, although the insulation effect was insignificant on the rainy day. Therefore, the type of climate should be considered when we selecting wall insulation materials. For example, in areas with a cloudy and rainy climate, no significant difference in the insulation performances of the four types of wall insulation material is identified.

### 4.3. Research Limitations

The STHIM used in this study can be embedded within concrete for direct long-term temperature measurement. Although the STHIM has a battery powered design, its peripheral circuit processing for signal transmission and reception consumes most of the electrical power. Considering the entire STHIM is embedded inside the RC structure of the building and that a building lifespan (50 year or more) is longer than the battery life of electronic products, an external power supply is required as a power source for the electronic circuitry. Additionally, it is impossible at this time to prove that a small battery could be used in a STHIM for ten or more years.

To address the issue of electricity consumption, controller power conservation and circuit design were examined. The nominal capacity of a CR 2450 battery is 660 mA/h; therefore, ten or more years could be obtained if the STHIM was suspended every 30 min [29]. When designing sensing circuits, the question of power consumption for each component must be considered and the component specifications must consider the theoretical value of the power consumption of each operational component to select components with minimal power consumption. The area where the overall circuit produces the greatest power consumption is the sensor transmission, especially during information transmission. Therefore, its transmission intervals must be considered and the theoretical power consumption of its transmission and suspend mode must be analyzed using the sensor specifications to select a suitable transmission interval based on the data. Future results of other electric power research teams will provide solutions to these power supply problems, such as an increase in small battery capacity, the maturity of external power charging technology, or the reduction in power consumption of electronic components.

### 4.4. Discussion

Future studies can continue improving STHIM performance by exploring issues such as the stability of signal transmission, antenna miniaturization design, power supply endurance, and the convenience of maintenance. If these problems are addressed, it will assist in the development of innovative sensing technology similar to the STHIM that can be used in the construction industry as a building material for smart buildings to measure and record long-term dynamic variations in temperature and humidity within building materials, building roofs, and walls. Additionally, when measured and recorded data values are obtained, statistical and comparative analysis can be performed for different environmental conditions, location characteristics, roof styles, wall styles, direction conditions, and height conditions using the temperature database. Smart buildings have already become a global trend and building self-health management is developing toward computerization and automation. Therefore, using plans that combine STHIM with a BPIS self-monitoring system can assist in future studies and the development of building self-diagnosis or self-repair capabilities (*i.e.*, leak location diagnosis or wall temperature automatic adjustment), allowing buildings to achieve the integrated performance of measurement, control, and management through the combination of sensors and control systems. Finally, we expect to improve the emitting and receiving efficiencies of antennas, increase the effectiveness wireless data collection from temperature and humidity sensors, and transmit effective data to BPIS through impedance matching designs in future studies.

## 5. Conclusions

This study tested the STHIM developed in five simulated roof samples and five sets of simulated wall samples. We successfully measured long-term temperature variations within concrete, proving that STHIM can overcome the adverse environment of concrete's strong alkalinity, water permeability, and pulp fluidity. We also observed in the two groups of experiments that the temperature variation inside concrete is influenced by insulation material performance, weather, absorption and radiation rates, direction, and solar radiation amount. Additionally, if an inappropriate insulation material is selected, it can result in temperatures within the concrete to be higher than when there is no insulation material. These results can provide building designers and property maintenance personnel with a reference for addressing energy conservation issues, emission reductions, and energy management.

## Figures and Tables

**Table 1.** Limitations of using common temperature sensing components.

**Details**		**Material**	**Measurement range**	**Common use**	**Limitations of the use in this study**
**Type**					
Thermocouple	K-type	Ni-Cr alloy, Al-Ni alloy	−200 °C to 1,200 °C	Industrial measurement of high temperature	The greatest inconvenience in measuring building structures is the requirement for zero corrections of the temperature and the signal noise ratio formed by small analog voltage and noise at the output, resulting in signal processing difficulty.
	E-type	Ni-Cr alloy, Cu	−100 °C to 800 °C		
	J-type	Fe, W, Cu	−200 °C to 750 °C		
	R-type	Pt, Rh	0 °C to 1,700 °C		
IC temperature sensor		Semiconductor	−50 °C to 150 °C	Home appliance temperature control	At high temperatures, the output voltage shows indicial response.
Resistance Temperature Detector (RTD)		Mn, Ni, Cu, Pt	−50 °C to 300 °C	Industrial use (*i.e.*, Pt-100)	To achieve accurate temperature measurement, the electrical circuit design for signal processing is complicated.
Thermistor	NTC	Sintered by Mn, Cu, Co, Fe, Ni	−100 °C to 450 °C	Electronic switches	High non-linearity and non-homogeneity is usually applicable for switch behavior and is not applicable for achieving effective temperature monitoring by the temperature analog signal process.
	PTC	Zn	−50 °C to 150 °C		
	CTR	Ceramics, V, Ba, CS, P	0 °C to 150 °C		

**Table 2.** Efficacy comparisons of roof thermal insulation materials.

	**R1-control group**	**R2-sod roof**	**R3-PS insulating brick**	**R4-painted corrugated sheet**	**R5-heat insulation paint**
**Sunny day sample maximum temperature (15:40)**	31.1 °C	22.6 °C	24.4 °C	27 °C	28.9 °C
**temperature difference between the R1**	-	8.5 °C	6.7 °C	4.1 °C	2.8 °C
**Cloudy day sample maximum temperature (15:15)**	29.9 °C	24.2 °C	25.5 °C	27.6 °C	28.3 °C
**temperature difference between the R1**	-	5.7 °C	4.4 °C	2.3 °C	1.6 °C
**Rainy day sample maximum temperature (15:05)**	30.8 °C	26.5 °C	27.3 °C	29.1 °C	29.8 °C
**temperature difference between the R1**	-	4.3 °C	3.5 °C	1.7 °C	1 °C

**Table 3.** Maximum temperature times inside simulated roof concrete samples.

	**R1-control group**	**R2-sod roof**	**R3-PS insulating brick**	**R4-painted corrugated sheet**	**R5-heat insulation paint**	**Atmosphere**
**Sunny day (11/24/2011) Maximum temperature**	31.1 °C	23.3 °C	25.3 °C	27.5 °C	28.9 °C	29.1 °C
**Sunny day (11/24/2011) Maximum temperature times**	15:40 to 16:25	17:50 to 19:20	17:35 to 19:05	16:25 to 17:15	15:30 to 16:25	13:35
**Sunny day (11/24/2011) Maximum temperature duration**	45 min	90 min	90 min	50 min	55 min	5 min

**Table 4.** Maximum Temperature Times Inside Simulated Wall Concrete samples.

	**W1-control group east side**	**W1-control group south side**	**W1-control group west side**	**W1-control group north side**	**Atmosphere**
**Sunny day (11/24/2011) Maximum temperature**	27 °C	37 °C	31.6 °C	24.3 °C	29.1 °C
**Sunny day (11/24/2011) Maximum temperature times**	15:05 to 15:50	14:45 to 15:25	16:30 to 16:50	15:45 to 17: 00	13:35
**Sunny day (11/24/2011) Maximum temperature duration**	45 min	40 min	20 min	75 min	5 min

**Table 5.** Efficacy comparison of outer-wall thermal insulation materials under different weather conditions.

	**W1-control group** **(south side)**	**W2-dark ceramic tile** **(south side)**	**W3-pale ceramic tile** **(south side)**	**W4-white paint** **(south side)**	**W5-stone** **(south side)**
**Sunny day sample maximum temperature (14:45)**	37 °C	37.6 °C	31.3 °C	25.7 °C	29.4 °C
**Temperature difference between the R1**	-	-0.6 °C	5.7 °C	11.3 °C	7.6 °C
**Cloudy day sample maximum temperature (13:15)**	32.5 °C	33.1 °C	29.2 °C	25.7 °C	28.4 °C
**Temperature difference between the R1**	-	−0.6 °C	3.3 °C	6.8 °C	4.1 °C
**Rainy day sample maximum temperature (13:35)**	31.2 °C	31.7 °C	29.7 °C	28 °C	29.1 °C
**Temperature difference between the R1**	-	-0.5 °C	1.5 °C	3.2 °C	2.1 °C
